# Effects of lactic acid bacteria-fermented dandelion *Jiangshui* on blood glucose levels in hyperglycemic mice

**DOI:** 10.3389/fmicb.2026.1910064

**Published:** 2026-08-13

**Authors:** Xian-Gang Meng, Jia-ni Lu, Hui Yao, Qing-kai Jin, Yi-xin Zhang, Zhi-hui Wang, Tuan-Jie Che, Yan-Wen Gui

**Affiliations:** 1 Department of Bioengineering, School of Biological and Pharmaceutical Engineering, Lanzhou Jiaotong University, Lanzhou, China; 2 Innovation Center of Functional Genomics and Molecular Diagnostics Technology of Gansu Province, Engineering Laboratory for Biochips of Gansu Province, Lanzhou, China

**Keywords:** blood glucose regulation, dandelion *Jiangshui*, inflammatory response, lactic acid bacteria, oxidative stress, pathological section

## Abstract

**Introduction:**

*Taraxacum mongolicum* Hand.-Mazz. is rich in dietary fiber and has been reported to exhibit hypoglycemic activity. This study investigated the effects of lactic acid bacteria (LAB)-fermented dandelion Jiangshui (JS) on glucose metabolism and pancreatic and renal histopathology in hyperglycemic mice.

**Methods:**

*Lactiplantibacillus plantarum* A1 and *Lacticaseibacillus paracasei* strains A3 and L2, previously isolated from traditional JS by our research team, were used to prepare dandelion JS by fermentation. Forty-five 5-week-old male C57BL/6 mice were randomly assigned to a normal control group, a model group, and a JS intervention group. Mice in the JS group received LAB-fermented dandelion JS by gavage, whereas mice in the other groups received an equal volume of normal saline for 14 consecutive weeks. Body weight, glucose-related biochemical indicators, and pancreatic and renal histopathology were monitored throughout the experiment.

**Results:**

Compared with the model group, the JS group showed attenuated increases in blood glucose, higher levels of the anti-inflammatory cytokine IL-10, increased hepatic antioxidant enzyme activities, including glutathione peroxidase (GSH-Px), superoxide dismutase (SOD), and catalase (CAT), lower malondialdehyde (MDA) content, and less severe pancreatic and renal lesions.

**Discussion:**

These findings suggest that LAB-fermented dandelion JS may have potential to improve glucose regulation and alleviate inflammation, oxidative stress, and pancreatic and renal injury in hyperglycemic mice.

## 1 Introduction

*Jiangshui* (JS) is a characteristic fermented food in northwest China that is naturally prepared using seasonal green vegetables, pre-prepared wheat flour soup, and mature JS starter, with fermentation driven mainly by microorganisms on the vegetable surface (Li et al., 2020). It is rich in nutrients and has traditionally been consumed as a cooling beverage, especially in summer. However, the traditional preparation of JS has drawbacks such as a long production cycle, short shelf life, and high susceptibility to microbial contamination. Current research on JS mainly focuses on strain isolation, with relatively limited studies on its functional properties based on the fermented substrate. Therefore, investigating the functional characteristics of JS is of great significance for extending its shelf life, improving product quality, and developing its functional applications.

Lactic acid bacteria (LAB) are the dominant microbial population in JS and play a key role in all stages of fermentation (Xu et al., 2024). Accumulating evidence has demonstrated the hypoglycemic effects of LAB, which can significantly delay the onset and progression of symptoms in diabetic patients, highlighting the important value of probiotics in the field of metabolic health (American Diabetes Association, 2024; Xu et al., 2024). Li et al. (2016) confirmed that the intake of *Lactiplantibacillus* plantarum CCFM0236 could significantly improve insulin resistance, enhance the antioxidant defense system, and reduce systemic inflammation in mice. *Lacticaseibacillus paracasei* has shown prominent performance in diabetes management: Dang et al. (2018) found that gavage of *Lacticaseibacillus paracasei* TD062 in diabetic mice improved fasting and postprandial blood glucose levels, enhanced glucose tolerance, and promoted hepatic glycogen accumulation and lipid metabolism (International Diabetes Federation, 2021). In addition, Parichart et al. (2020) reported that the intake of *Lacticaseibacillus paracasei* HII01 in diabetic rats ameliorated intestinal microecological disorders, alleviated intestinal barrier damage, relieved endotoxemia and insulin signaling disorders, and effectively regulated blood glucose levels.

*Taraxacum mongolicum Hand.-Mazz*., an edible and medicinal plant (Wan et al., 2023), has great potential for the treatment of metabolic diseases due to its diverse functional components (Li X. S. et al., 2022; Wu et al., 2022; Li et al., 2025). Studies have shown that dandelion is rich in medicinal compounds with hypoglycemic and hypolipidemic effects (Zhou et al., 2023), as well as functional ingredients such as dandelion polysaccharides and organic acids, which exert antioxidant and immunomodulatory effects (Wang et al., 2021). Dandelion and its products also exhibit positive therapeutic effects in the treatment of gastrointestinal diseases (Li Y. N. et al., 2022).

Previous studies have generally examined probiotic preparations and dandelion-derived products as separate interventions. Multi-strain probiotics improved glycemic regulation in diabetic mice through modulation of gut microbiota, short-chain fatty acid production, and GLP-1 secretion (Wang et al., 2020; Ahmad et al., 2023). Dandelion research has mainly focused on unfermented extracts: dandelion water extract reduced serum glucose and hepatic lipid peroxidation in STZ-induced diabetic rats (Cho et al., 2002), while recent research characterized the enzyme-inhibitory and antioxidant potential of dandelion root extracts *in vitro* (Zolotova et al., 2024). However, relatively limited information is available on the metabolic effects of a whole dandelion Jiangshui matrix fermented with a defined consortium of Jiangshui-derived LAB.

Therefore, the present study prepared LAB-fermented dandelion JS via inoculated fermentation and evaluated its effects in C57BL/6 mice with HFD/STZ-induced hyperglycemia. The aim of this study was to investigate the effects of LAB-fermented dandelion JS on glucose-related indicators, inflammation, oxidative stress, pancreatic and renal histopathology, and gut microbiota. This integrated design was intended to provide preliminary evidence for the development of LAB-fermented dandelion JS as a functional fermented food.

## 2 Materials and methods

### 2.1 Materials and reagents

*Taraxacum mongolicum Hand.-Mazz*. was collected from a planting base in *Wusha*n County, Gansu Province, China; household wheat flour (Gan-qing universal flour) was commercially purchased. *Lactiplantibacillus plantarum A1, Lacticaseibacillus paracasei A3, and Lacticaseibacillus paracasei L2* were previously isolated and screened from naturally fermented JS of *Taraxacum mongolicum* and maintained under laboratory culture conditions. Forty-five specific pathogen-free (SPF) 5-week-old male C57BL/6 mice were purchased from Gansu Lanzhou Veterinary Research Institute (License No.: SCXK 2020–0002). MRS medium, streptozotocin (STZ), hematoxylin staining solution, and eosin staining solution were purchased from Solar bio-Sci & Tech Co., Ltd. (Beijing, China). Iodine, potassium iodide, glucose, concentrated hydrochloric acid, sodium hydroxide, and phenolphthalein were obtained from commercial suppliers (Guangdong Guanghua Sci-Tech Co., Ltd., Shanghai Macklin Biochemical Co., Ltd., Yantai SS Chemical Co., Ltd., Sinopharm Chemical Reagent Co., Ltd., Shandong AC Biotechnology Co., Ltd., respectively). ELISA kits for glycosylated hemoglobin (HbA1c), insulin, endotoxin, hepatic glycogen, tumor necrosis factor-*α* (TNF-α), interleukin (IL)-1β, IL-6, IL-10, GSH-Px, SOD, MDA, and CAT were purchased from Wuhan Bionline Biotechnology Co., Ltd. (Wuhan, China). Xylene, absolute ethanol, 95% ethanol, and neutral gum were obtained from Tianjin HX Chemical Reagent Manufacturing Co., Ltd. and Shanghai Specimen Model Factory, respectively.

### 2.2 Instruments and equipment

Electronic balance (Shanghai Shunyu Hengping Scientific Instrument Co., Ltd.); high-speed desktop centrifuge (Shanghai Anting Scientific Instrument Factory); pipettes (Eppendorf, Germany); vortex mixer (Haimen Qilin Bell Instrument Manufacturing Co., Ltd.); pH meter (Dongguan Wanchuang Electronic Products Co., Ltd.); digital display constant temperature water bath (Shanghai Lichen Bangxi Instrument Technology Co., Ltd.); UV–visible spectrophotometer (Shanghai Yuan-xi Instrument Co., Ltd.); liquid chromatography-mass spectrometry system (Agilent Technologies, United States); SPX intelligent biochemical incubator (Ningbo Southeast Instrument Co., Ltd.); 101-1AB electric blast drying oven (Tianjin Tai-site Instrument Co., Ltd.); MB16-414 microplate reader (Shanghai Jing-ke Instrument Co., Ltd.); embedding machine (Jinhua Kedi Instrument Co., Ltd., Zhejiang Province); microtome (LEICA2016, Germany); optical microscope (Leica DM2500, Germany).

### 2.3 Experimental methods

#### 2.3.1 Preparation of LAB-fermented dandelion JS

LAB-fermented dandelion JS was prepared by single-strain and mixed-strain inoculation using the preserved strains. Washed dandelion leaves were cut into 2–3 cm segments, blanched in boiling water for 15–30 s, and then cooled. Wheat starchy soup was prepared by adding wheat flour at 1% (w/v) of the total water volume, boiled, and cooled to below 40 °C. Cooled dandelion and starchy soup were added to a beaker at 14% (v/v) and 50% (v/v) of the total preparation volume, respectively, sterilized at 121 °C for 20 min, and cooled to room temperature. The sterilized mixture was inoculated with a 36% (v/v) bacterial suspension with an OD_600_ value of 0.5–0.7. The fermentation treatments were as follows: single-strain inoculation (Treatment 1: A1; Treatment 2: A3; Treatment 3: L2) and mixed-strain inoculation (Treatment 4: A1: A3 = 1:1, v/v; Treatment 5: A1: L2 = 1:1, v/v; Treatment 6: A3: L2 = 1:1, v/v; Treatment 7: A1: A3: L2 = 1:1:1, v/v). The mixtures were sealed and fermented at 28 °C for 36 h (fermentation maturity). The pH value, total acid content, and reducing sugar content were determined every 12 h during fermentation. The pH value was measured using a pH meter; reducing sugar content was determined by the DNS method; total acid content was measured by acid–base titration. The viable LAB count at fermentation maturity was determined by plate counting on MRS agar.

#### 2.3.2 Sensory evaluation

A sensory evaluation panel consisting of 10 healthy teachers and students with no adverse habits, dietary preferences, or allergic reactions was established. The sensory evaluation of hypoglycemic LAB-fermented dandelion JS was performed according to the national standard GB/T 10220–2012 with appropriate modifications based on the method of Xu et al. (2024). A 100-point scoring system was adopted, and the evaluation criteria included turbidity (25 points), color (25 points), aroma (25 points), and taste (25 points) (Table 1).

**Table 1:** Sensory evaluation criteria.

Sensory index	Scoring criteria			
	Level 1	Level 2	Level 3	Level 4
Turbidity (25 points)	No precipitation, clear (19–25)	Slight precipitation, slightly turbid (13–18)	More precipitation, relatively turbid (7–12)	Severe precipitation, severely turbid (1–6)
Color (25 points)	Bright color, uniform color (19–25)	Relatively dark color, uniform color (13–18)	Relatively dark color, locally uneven color (7–12)	Dark color, uneven color (1–6)
Aroma (25 points)	Fragrant smell, mild sour taste, no peculiar smell (19–25)	Relatively light aroma, no peculiar smell (13–18)	Very light aroma, slightly peculiar smell (7–12)	No aroma, strong peculiar smell (1–6)
Taste (25 points)	Moderate acidity, soft taste (19–25)	General acidity (13–18)	Relatively light sour taste (7–12)	No sour taste, poor taste (1–6)

Sensory evaluation was conducted by a 10-member panel. Each sensory attribute was scored on a 25-point scale, with a maximum total score of 100. Higher scores indicate better sensory quality.

#### 2.3.3 LC–MS analysis of selected phenolic compounds

Samples collected before fermentation (0 h) and at fermentation maturity (36 h) were analyzed by LC–MS. Briefly, 0.1 mL of sample was extracted with 0.9 mL of 70% methanol, centrifuged, and filtered through a 0.22-μm membrane. Separation was performed on an Agilent C18 column (2.1 × 100 mm, 1.8 μm) at 30 °C with a flow rate of 0.3 mL/min and an injection volume of 2 μL, using 0.1% formic acid in water and acetonitrile as the mobile phases under gradient elution. Data were acquired in positive and negative electrospray ionization modes, and the target compounds were quantified and expressed as μg/L.

#### 2.3.4 Establishment and grouping of experimental animal models

Forty-five 5-week-old male C57BL/6 mice were raised in the SPF animal house of Building 22, China Western Science and Technology Innovation Port, Xi’an New District, Shaanxi Province. The animal house was maintained at 22 ± 2 °C with a relative humidity of 60% and a 12 h light/dark cycle. Mice had free access to food and water throughout the experiment.

A preventive gavage method was adopted in this study, in which JS intervention was administered simultaneously with model establishment. The diabetic model was established by a high-sugar and high-fat diet combined with low-dose STZ injection: STZ was dissolved in 0.1 mol/L citric acid buffer (pH 4.5) and prepared fresh for use. Mice were intraperitoneally injected with STZ at a dose of 60 mg/kg body weight once daily for 3 consecutive days (Ma et al., 2020). Mice in the blank control group were injected with 0.85% (w/v) normal saline instead of STZ solution. All mice were fed a basal diet (12% fat, 65% carbohydrate, 22% protein) for a 1-week adaptation period, and then randomly divided into three groups with 15 mice in each group: normal control group (N), model group (M), and JS group (J). Body weight was measured weekly, and food intake was recorded throughout the experiment. Except for the normal control group, which was continuously fed the basal diet, the M and J groups received a high-sugar and high-fat diet consisting of 67% basal diet, 10% lard, 20% sucrose, 2.5% cholesterol, and 0.5% sodium cholate, expressed as weight percentages of the final diet (w/w) (Li et al., 2018). Mice in the J group were gavaged once daily with JS at a dose of 0.1 mL/10 g body weight, corresponding to approximately 200–300 μL per mouse according to body weight. The administered JS contained 6.67 × 10^8^ CFU/mL viable LAB, while mice in the N and M groups received an equal volume of 0.9% (w/v) normal saline (Table 2). Model establishment started from the 2nd week and lasted for 6 weeks. The diabetic model was considered successful when the fasting blood glucose level was higher than 11.1 mmol/L.

**Table 2:** Grouping of experimental animals.

Groups	Week 2 ~ 4	Week 5	Week 6 ~ 14
N	Basal diet + Normal saline (0.1 mL/10 g body weight)	Basal diet + intraperitoneal injection of normal saline + Normal saline (0.1 mL/10 g body weight)	Basal diet + Normal saline (0.1 mL/10 g body weight)
M	High-sugar and high-fat diet + Normal saline (0.1 mL/10 g body weight)	High-sugar and high-fat diet + intraperitoneal injection of STZ + Normal saline (0.1 mL/10 g body weight)	High-sugar and high-fat diet + Normal saline (0.1 mL/10 g body weight)
J	High-sugar and high-fat diet + JS (0.1 mL/10 g body weight)	High-sugar and high-fat diet + intraperitoneal injection of STZ + JS (0.1 mL/10 g body weight)	High-sugar and high-fat diet + JS (0.1 mL/10 g body weight)

N, normal control group receiving the basal diet and normal saline; M, HFD/STZ-induced hyperglycemic model group receiving normal saline; J, HFD/STZ-induced hyperglycemic group receiving LAB-fermented dandelion Jiangshui. STZ, streptozotocin; JS, LAB-fermented dandelion Jiangshui.

#### 2.3.5 Collection and processing of tissue samples

Tissue sample collection and processing were performed according to the method of Yu et al. (2017) with minor modifications. At 6, 10, and 14 weeks of feeding, 5 mice in each group were sacrificed. Mice were fasted overnight with free access to water before sacrifice. Whole blood was collected by enucleation, allowed to stand, and centrifuged at 4000 r/min for 8 min at 4 °C to separate serum, which was stored at −80 °C for subsequent use. Mice were sacrificed by cervical dislocation, and pancreatic and renal tissues were immediately dissected. A portion of the liver tissue was homogenized with pre-cooled normal saline to prepare a 10% (w/v) liver homogenate, which was centrifuged at 4000 r/min for 8 min at 4 °C; the supernatant was collected and stored at −80 °C for use. Pancreatic and renal tissues were fixed in 4% (v/v) formaldehyde solution for pathological examination.

#### 2.3.6 Index measurement

##### 2.3.6.1 Determination of body weight and fasting blood glucose concentration

Body weight was measured once a week, and fasting blood glucose concentration was determined every 2 weeks during the experiment. Mice were fasted for 12 h with free access to water before blood glucose measurement.

##### 2.3.6.2 Oral glucose tolerance test

At the end of the experiment, mice were fasted overnight and orally gavaged with a 30% (w/v) glucose solution at a dose of 2 g/kg body weight (Bartoli et al., 2011). Blood glucose levels were measured at 0, 30, 60, 90, and 120 min after glucose administration.

##### 2.3.6.3 Determination of diabetes-related biochemical indicators

Serum HbA1c and insulin levels, as well as hepatic glycogen content in liver homogenate, were determined by ELISA according to the manufacturer’s instructions for the corresponding kits.

##### 2.3.6.4 Determination of serum inflammatory factors

Serum levels of TNF-*α*, IL-1β, IL-6, and IL-10 were measured by ELISA following the kit instructions.

##### 2.3.6.5 Determination of hepatic oxidative stress indicators

The levels of GSH-Px, SOD, MDA, and CAT in liver homogenate stored at −80 °C were determined by ELISA according to the kit protocols.

##### 2.3.6.6 Pathological section observation of pancreas and kidney

Formalin-fixed pancreatic and renal tissues were washed, trimmed, dehydrated with gradient ethanol, cleared with xylene, embedded in paraffin, and sectioned into 5 μm-thick slices using a microtome. The sections were stained with hematoxylin–eosin (HE), dehydrated, and mounted with neutral gum. Pathological changes were observed under an optical microscope at 200 × magnification, and images were collected.

#### 2.3.7 Gut microbiota analysis

Gut microbiota analysis was performed according to Ai et al. (2021), with minor modifications. At week 14, fresh fecal samples were collected individually from five mice per group. Microbial genomic DNA was extracted from 15 fecal samples using the E. Z. N. A. Mag-Bind Soil DNA Kit (Omega Bio-tek, Norcross, GA, United States). The V3–V4 region of the bacterial 16S rRNA gene was amplified using primers 338F (5′-ACTCCTACGGGAGGCAGCAG-3′) and 806R (5′-GGACTACHVGGGTWTCTAAT-3′). PCR conditions were 95 °C for 3 min; 27 cycles of 95 °C for 30 s, 55 °C for 30 s, and 72 °C for 45 s; followed by 72 °C for 10 min. Purified amplicons were pooled and subjected to paired-end sequencing on an Illumina platform. Raw reads were quality-filtered using fastp v0.23.4 and merged using FLASH v1.2.11. High-quality sequences were clustered into operational taxonomic units at 97% similarity using UPARSE, and chimeras were removed using UCHIME. Taxonomic assignment was performed using the RDP classifier against SILVA 138.1 at a confidence threshold of 70%. Chao1, Shannon, and Simpson indices were calculated in Mothur, and bacterial relative abundances were summarized at the phylum and genus levels.

#### 2.3.8 Statistical analysis

All experimental data were expressed as the mean ± standard deviation (x ± s). Data analysis was performed using SPSS 22.0 software, and graphing was completed with Origin 2021 and Microsoft Office software. One-way analysis of variance (ANOVA) with the least significant difference (LSD) test was used for intergroup comparisons. A *p* < 0.05 was considered statistically significant. Pathological data were analyzed by comparative descriptive analysis.

## 3 Results and analysis

### 3.1 Sensory scores of LAB-fermented dandelion JS

**Figure 1:**
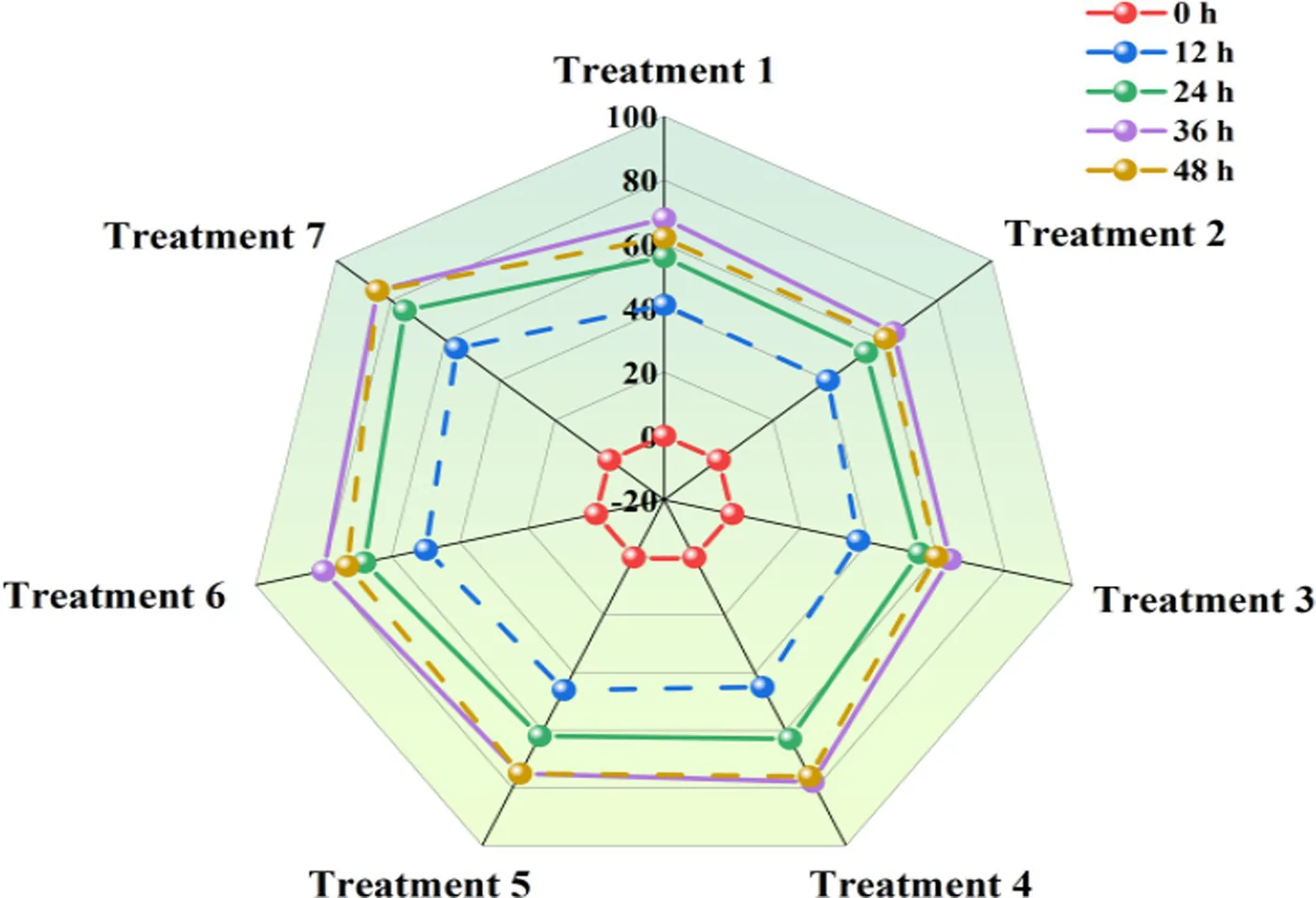
Sensory scores of dandelion Jiangshui subjected to different fermentation treatments during fermentation. Sensory evaluation was performed at 0, 12, 24, 36, and 48 h based on turbidity, color, aroma, and taste. The radial axis represents the sensory score, and the angular axes represent the fermentation treatments (T1–T7). Each value represents the mean score obtained from a 10-member sensory panel.

Figure 1 shows the sensory scores of the different fermentation treatments. T7 (A1: A3: L2 = 1:1:1, v/v/v) received the highest sensory score and was characterized by no precipitation, a clear appearance, bright and uniform color, fresh aroma without off-flavor, and a mild taste with moderate acidity. Therefore, dandelion JS fermented with A1, A3, and L2 at a ratio of 1:1:1 (v/v/v) was selected for the subsequent animal experiments. At fermentation maturity (36 h), the viable LAB concentration in the product used for gavage was 6.67 × 10^8^ CFU/mL.

### 3.2 Changes in selected phenolic compounds during fermentation

LC–MS analysis showed that the detectable phenolic composition of dandelion Jiangshui changed after 36 h of fermentation (Table 3). Chlorogenic acid, caffeic acid, isochlorogenic acid A, and isochlorogenic acid C were not detected at 0 h but were detected at 36 h at concentrations of 1,158.46, 190.96, 140.70, and 164.60 μg/L, respectively. Luteoloside increased from 52.59 to 89.33 μg/L, and chlorogenic acid was the most abundant of the five selected compounds in the fermented sample.

**Table 3:** Changes in selected phenolic compounds in dandelion Jiangshui during LAB fermentation.

Compound	0 h (μg/L)	36 h (μg/L)
Chlorogenic acid	ND	1158.46
Caffeic acid	ND	190.96
Isochlorogenic acid A	ND	140.70
Isochlorogenic acid C	ND	164.60
Luteoloside	52.59	89.33

Samples at 0 and 36 h represent dandelion Jiangshui before fermentation and at fermentation maturity, respectively. ND, not detected.

### 3.3 Effect on body weight of diabetic mice

**Figure 2:**
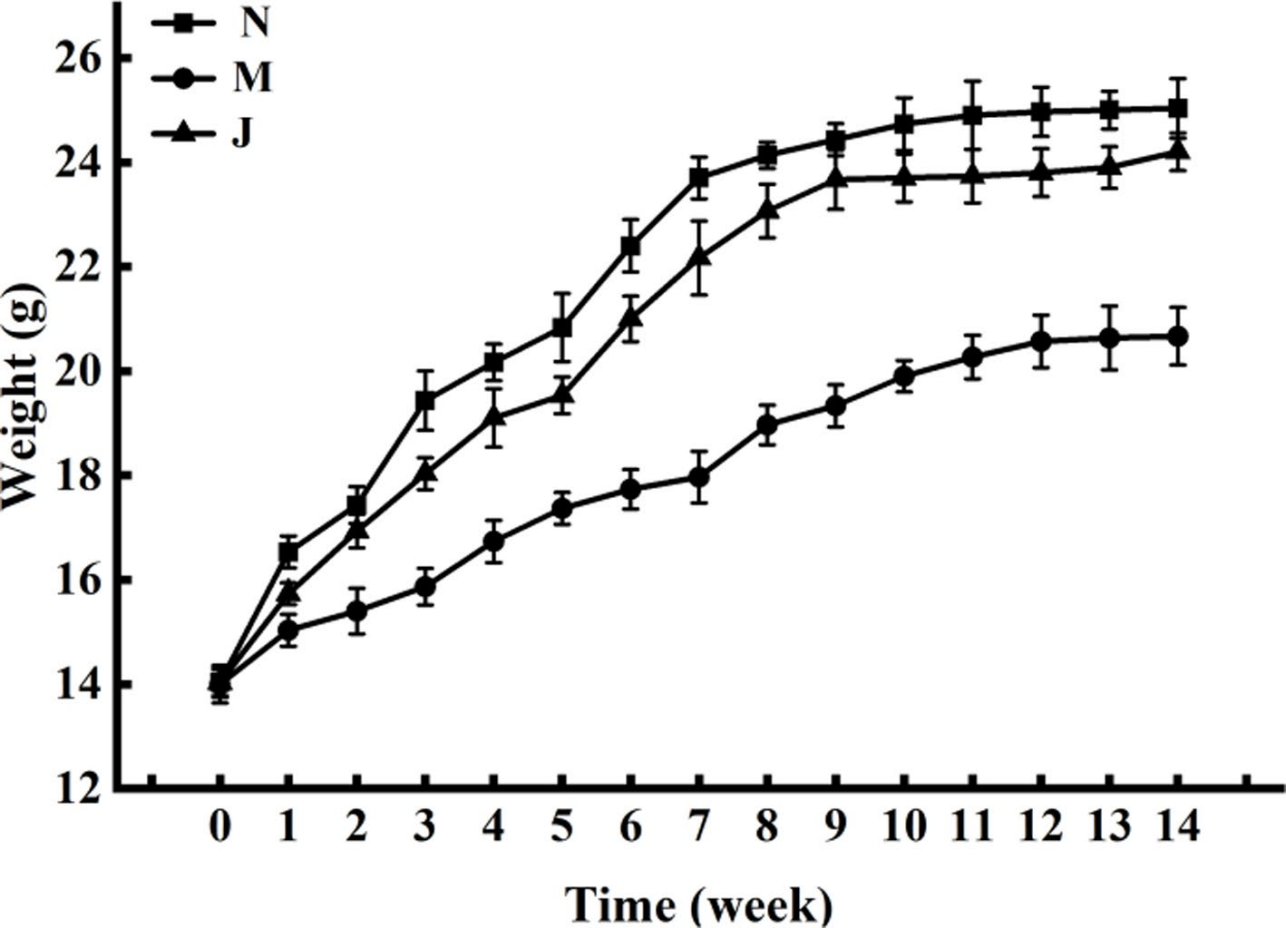
Body weight changes in mice during the 14-week intervention period. *N*, normal control group receiving the basal diet and normal saline; *M*, HFD/STZ-induced hyperglycemic model group receiving normal saline; *J*, HFD/STZ-induced hyperglycemic group receiving LAB-fermented dandelion Jiangshui. Data are presented as the mean ± SD. Sample sizes were *n* = 15 mice per group at weeks 0–6, *n* = 10 at weeks 7–10, and *n* = 5 at weeks 11–14 because of scheduled sampling. Group differences at each time point were analyzed using one-way ANOVA followed by the LSD test, with *p* < 0.05 considered statistically significant.

As shown in Figure 2, mice were randomly divided into three groups before the experiment, so the initial body weight of mice in each group was basically consistent. After 14 weeks of feeding, the body weight changes of the three groups were measured. The results showed that the body weight of mice in all three groups increased, but the weight gain rate varied among groups. Mice in the N group had the highest weight gain rate with the most significant body weight change; the weight gain of mice in the M group was significantly lower than that in the N group (*p* < 0.05), which may be due to pathological changes in the body caused by long-term intake of a high-sugar and high-fat diet, leading to decreased food intake and ultimately slow weight gain. Compared with the M group, the weight gain rate of mice in the J group was increased, indicating that the intervention of LAB-fermented dandelion JS improved the pathological state of diabetic mice. There was no significant difference in the weight gain rate between the J group and the N group (*p* > 0.05).

### 3.4 Effect on fasting blood glucose concentration of diabetic mice

According to the results in Table 4, a long-term high-sugar and high-fat diet led to a continuous increase in fasting blood glucose concentration in mice. At the 14th week, the fasting blood glucose concentration of mice in the M group reached 12.46 mmol/L, which was significantly higher than that of the N group (4.42 mmol/L, *p* < 0.05). After gavage with JS, the fasting blood glucose concentration of mice in the J group showed a downward trend, and the increase in blood glucose was significantly inhibited at the 10th week. At the 14th week, the fasting blood glucose concentration of the J group was 9.11 mmol/L, which was significantly different from that of the M group (*p* < 0.05). The above results indicated that LAB-fermented dandelion JS had a significant effect on reducing fasting blood glucose concentration in diabetic mice and a certain therapeutic effect on hyperglycemia symptoms.

**Table 4:** Effect of hypoglycemic LAB-fermented dandelion *Jiangshui* on fasting blood glucose in diabetic murine models.

Groups	Fasting blood glucose concentration/(mmol/L)				
	6 weeks	8 weeks	10 weeks	12 weeks	14 weeks
N	3.56 ± 0.51^b^	4.73 ± 0.48^c^	5.23 ± 0.55^c^	6.68 ± 0.43^c^	4.42 ± 0.45^c^
M	7.11 ± 1.05^a^	7.72 ± 1.45^a^	10.86 ± 0.75^a^	11.8 ± 1.24^a^	12.46 ± 1.87^a^
J	7.42 ± 0.26^a^	8.31 ± 0.68^b^	8.18 ± 0.66^b^	9.24 ± 0.46^b^	9.11 ± 0.33^b^

N, normal control group receiving the basal diet and normal saline; M, HFD/STZ-induced hyperglycemic model group receiving normal saline; J, HFD/STZ-induced hyperglycemic group receiving LAB-fermented dandelion Jiangshui. Data are presented as the mean ± SD. Sample sizes were *n* = 15 mice per group at week 6, *n* = 10 at weeks 8 and 10, and *n* = 5 at weeks 12 and 14 because of scheduled sampling. Different lowercase letters in the same column indicate significant differences among groups (one-way ANOVA followed by the LSD test, *p* < 0.05).

### 3.5 Effects on the oral glucose tolerance of hyperglycemic mice

As shown in Table 5, the fasting blood glucose level of mice in group M (12.28 mmol/L) was significantly higher than that of group N (7.64 mmol/L, *p* < 0.05), indicating that the diabetic animal model was successfully established using a high-sugar and high-fat diet combined with STZ injection. The blood glucose levels of mice in all groups peaked at 30 min after glucose gavage and then began to decrease, but the glucose concentration in group M remained at a high level. The blood glucose of mice in group N almost returned to the pre-administration level at 120 min after gavage, indicating normal oral glucose tolerance (Wen et al., 2018). The blood glucose level in group M reached 20.32 mmol/L at 120 min, which was much higher than the blood glucose level at 0 min, indicating that mice in this group basically lost the ability to regulate blood glucose. The blood glucose level in group J at 120 min was only slightly higher than the value at 0 min and was significantly lower than that in group M (*p* < 0.05). These results fully demonstrated that gavage of JS could effectively inhibit postprandial blood glucose elevation in mice and significantly improve their blood glucose regulation ability.

**Table 5:** Effect of hypoglycemic lab-fermented dandelion JS on oral glucose tolerance in diabetic murine models.

Groups	Blood glucose concentration/(mmol/L)				
	0 min	30 min	60 min	90 min	120 min
N	7.64 ± 0.66^b^	11.41 ± 1.52^c^	9.97 ± 2.34^c^	8.67 ± 2.11^c^	6.83 ± 2.48^c^
M	12.28 ± 1.65^a^	28.82 ± 2.16^a^	25.04 ± 3.34^a^	23.66 ± 2.65^a^	20.32 ± 2.75^a^
J	11.78 ± 1.33^a^	18.87 ± 3.65^b^	16.95 ± 2.77^b^	14.32 ± 3.36^b^	12.88 ± 1.88^b^

N, normal control group receiving the basal diet and normal saline; M, HFD/STZ-induced hyperglycemic model group receiving normal saline; J, HFD/STZ-induced hyperglycemic group receiving LAB-fermented dandelion Jiangshui. Data are presented as the mean ± SD (*n* = 5 mice per group). Different lowercase letters in the same column indicate significant differences among groups (one-way ANOVA followed by the LSD test, *p* < 0.05).

### 3.6 Effects on the diabetes-related indicators of hyperglycemic mice

HbA1c is a product of the combination of hemoglobin in red blood cells and blood glucose, which can reflect the blood glucose level in a specific period, and is used as an important evaluation index for diabetes together with insulin. The higher the blood glucose concentration, the higher the HbA1c content. As shown in Table 6, the relative content of HbA1c in group M (25.28 ± 3.11) was significantly higher than that in group N (13.21 ± 2.78, *p* < 0.05). Under hyperglycemic conditions, the serum endotoxin level in group M was significantly higher than that in group N, thus destroying the normal glucose metabolism mechanism of mice, inducing insulin resistance, and leading to an abnormal increase in insulin level in the body. This accelerated the decomposition and consumption of glycogen in the body, resulting in lower hepatic glycogen content in group M than in group N, further exacerbating glucose metabolism disorders.

**Table 6:** Effect of JS on diabetes-associated biochemical parameters in diabetic murine models.

Groups	Relative content of glycosylated hemoglobin	Insulin content/(μIU/mL)	Endotoxin content/(EU/mL)	Glycogen content in liver/(mg/g)
N	13.21 ± 2.78^b^	8.95 ± 1.32^a^	0.24 ± 0.03^b^	2.41 ± 1.04^a^
M	25.28 ± 3.11^a^	11.78 ± 3.12^a^	0.37 ± 0.03^a^	0.81 ± 0.19^b^
J	18.69 ± 5.88^ab^	10.06 ± 5.81^a^	0.29 ± 0.02^b^	2.01 ± 0.71^ab^

N, normal control group receiving the basal diet and normal saline; M, HFD/STZ-induced hyperglycemic model group receiving normal saline; J, HFD/STZ-induced hyperglycemic group receiving LAB-fermented dandelion Jiangshui. Data are presented as the mean ± SD (*n* = 5 mice per group). Different lowercase letters in the same column indicate significant differences among groups (one-way ANOVA followed by the LSD test, *p* < 0.05).

Compared with group M, the HbA1c content of mice in group J was significantly reduced after gavage with JS (*p* < 0.05), and the hepatic glycogen content increased to 2.01 mg/g, which was higher than 0.81 mg/g in group M. These results indicated that gavage of JS had a positive effect on improving diabetes-related biochemical indicators in mice and could alleviate the symptoms of blood glucose elevation and glucose metabolism disorder to a certain extent.

### 3.7 Serum inflammatory factors

**Figure 3:**
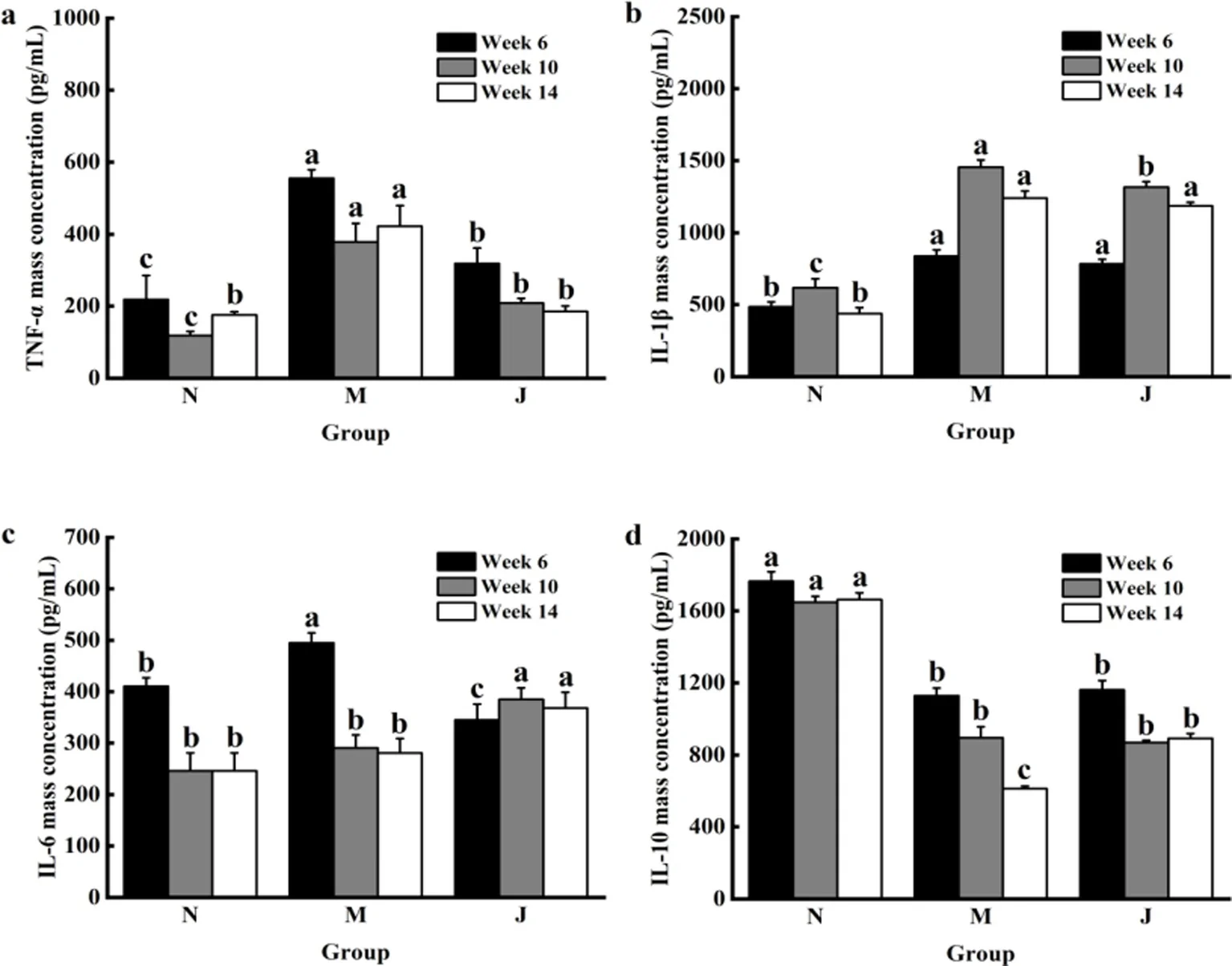
Effect of LAB-fermented dandelion Jiangshui on serum inflammatory cytokine levels in hyperglycemic mice. **(a)** TNF-*α*; **(b)** IL-1β; **(c)** IL-6; and **(d)** IL-10. N, normal control group receiving the basal diet and normal saline; M, HFD/STZ-induced hyperglycemic model group receiving normal saline; J, HFD/STZ-induced hyperglycemic group receiving LAB-fermented dandelion Jiangshui. Serum cytokine levels were measured at weeks 6, 10, and 14. Data are presented as the mean ± SD (*n* = 5 mice per group at each time point). Different lowercase letters at the same time point indicate significant differences among groups (*p* < 0.05; one-way ANOVA followed by the LSD test).

As shown in Figure 3, at 6, 10, and 14 weeks, the serum levels of pro-inflammatory factors TNF-*α*, IL-1β, and IL-6 in group M were higher than those in group N, with a significant difference between the two groups at 6 weeks (*p* < 0.05). In addition, the serum level of the anti-inflammatory factor IL-10 in group M was lower than that in group N at all three time points. Compared with group M, the serum IL-6 level in group J decreased at 6 weeks; the serum levels of TNF-α and IL-6 in group J increased significantly at 10 weeks but decreased again at 14 weeks. The serum IL-10 level in group J showed an increasing trend at 6, 10, and 14 weeks, and was significantly higher than that in group M at 14 weeks (*p* < 0.05). These results indicated that JS gavage may promote an anti-inflammatory response to hyperglycemia-associated inflammation.

### 3.8 Antioxidant indexes of liver homogenate

**Figure 4:**
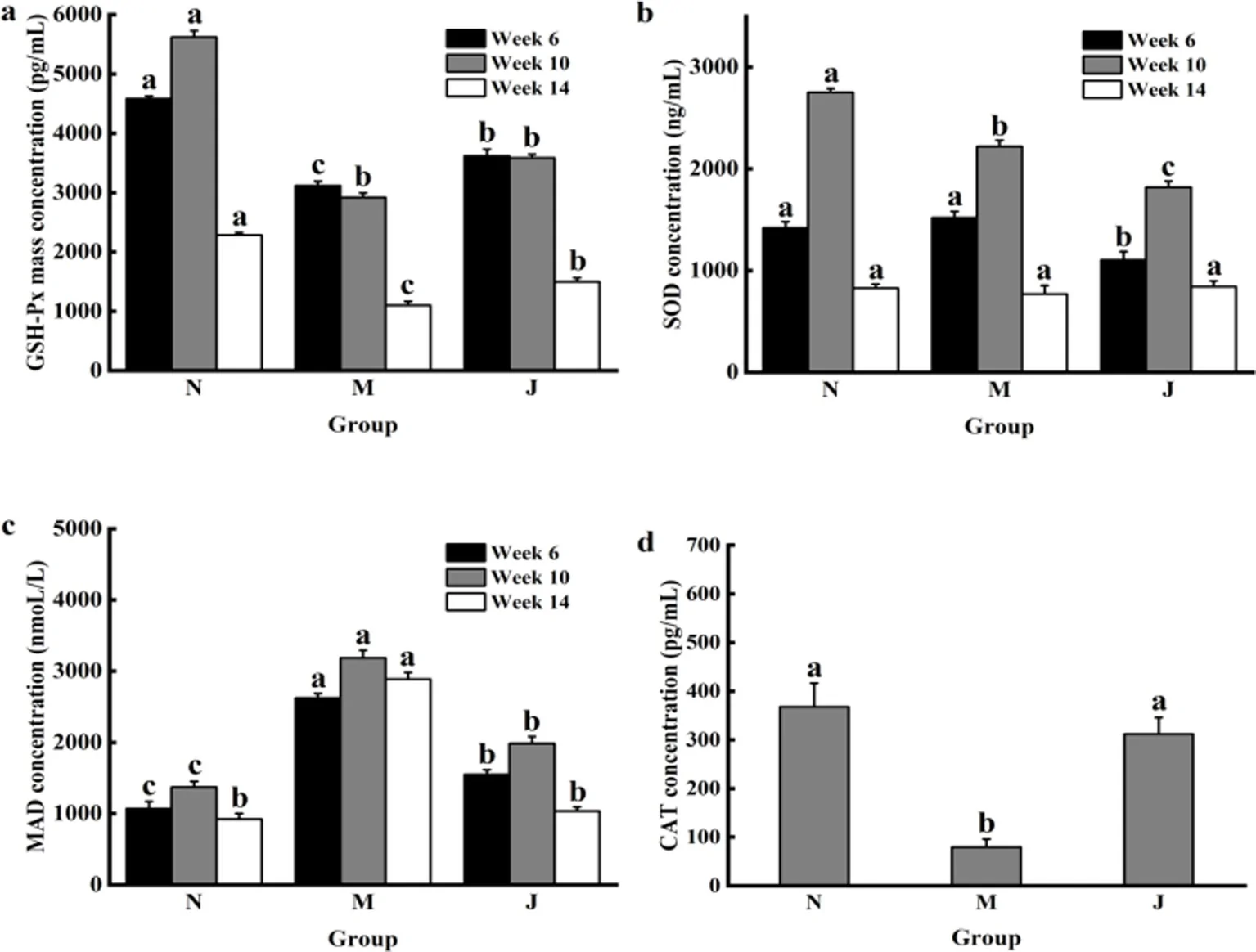
Hepatic oxidative stress-related indicators in mice. **(a)** GSH-Px; **(b)** SOD; **(c)** MDA; **(d)** CAT. Panels A–C show data at weeks 6, 10, and 14, whereas CAT in panel D was measured at week 14 only. N, normal control group receiving the basal diet and normal saline; M, HFD/STZ-induced hyperglycemic model group receiving normal saline; J, HFD/STZ-induced hyperglycemic group receiving LAB-fermented dandelion Jiangshui. Data are presented as mean ± SD (*n* = 5 mice per group at each indicated time point). Different lowercase letters at the same time point indicate significant differences among groups (one-way ANOVA followed by the LSD test, *p* < 0.05).

As shown in Figure 4, compared with group N, continuous feeding with a high-sugar and high-fat diet in group M led to a significant increase in hepatic MDA content (*p* < 0.05) and a decrease in GSH-Px and SOD activities, with a particularly significant difference in GSH-Px activity at 14 weeks (*p* < 0.05). The CAT activity of mice in group J decreased at 14 weeks (data at 6 and 10 weeks not shown), but there was no significant difference compared with group N (*p* > 0.05). These results indicated that long-term consumption of the high-sugar and high-fat diet could significantly increase the metabolic burden of the liver and cause severe oxidative damage to liver tissue. In mice in group J gavaged with JS, the hepatic antioxidant capacity was improved: GSH-Px activity was the most significantly enhanced, which was higher than that in group M at all three time points; SOD activity was lower than that in the M group at 6 weeks but gradually increased and exceeded that in group M at 14 weeks (no significant difference, *p* > 0.05); at 14 weeks, the CAT activity in group J was significantly higher than that in group M (*p* < 0.05), and the MDA level was significantly lower (*p* < 0.05). These results suggested that JS could effectively reduce the degree of hepatic peroxidation and enhance the antioxidant activity in diabetic mice.

### 3.9 Pathological changes of pancreatic and renal tissues

**Figure 5:**
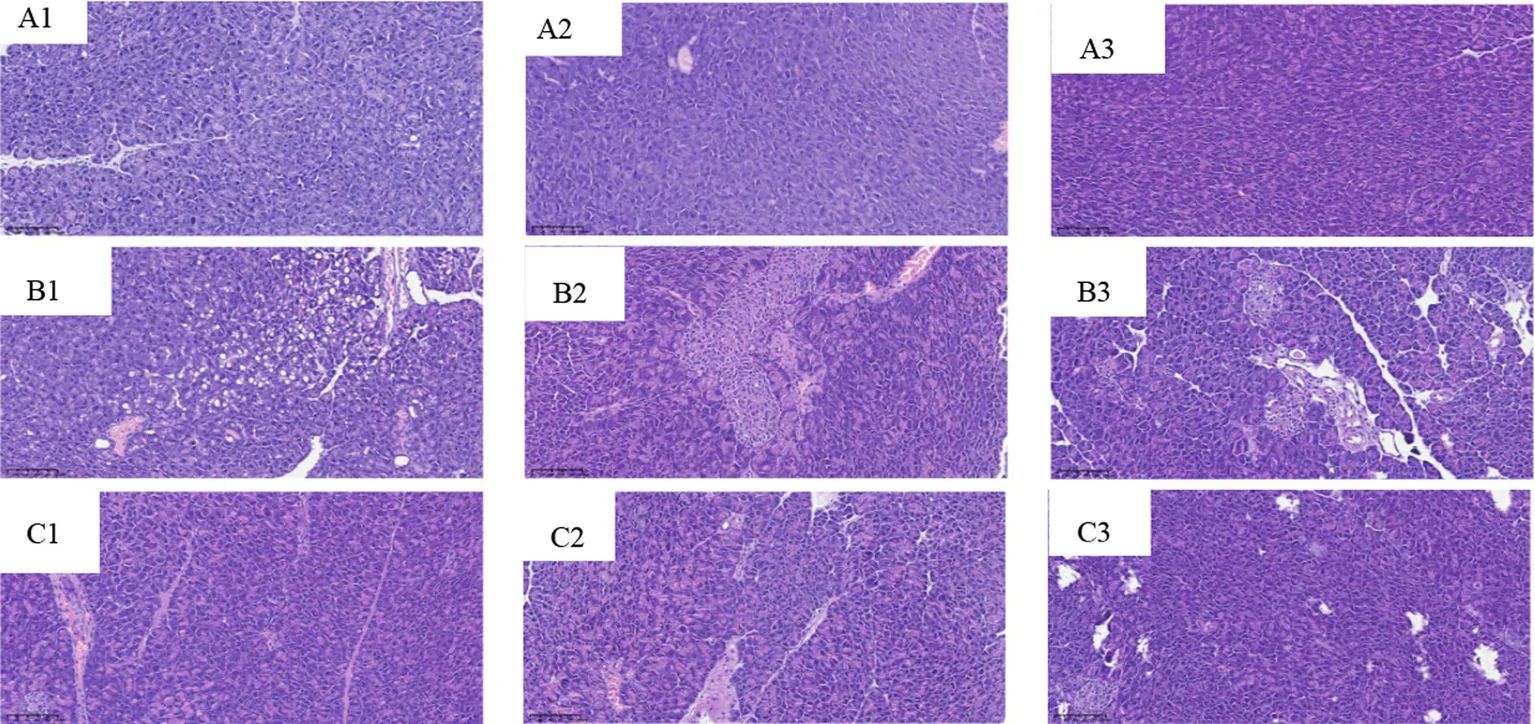
Representative H&E-stained pancreatic sections at weeks 6, 10, and 14 (200×). N, normal control group receiving the basal diet and normal saline; M, HFD/STZ-induced hyperglycemic model group receiving normal saline; J, HFD/STZ-induced hyperglycemic group receiving LAB-fermented dandelion Jiangshui. A, B, and C denote N, M, and J, respectively; subscripts 1–3 denote weeks 6, 10, and 14.

The H&E staining results in Figure 5 showed that pancreatic tissue in group N remained clear and intact, with regularly arranged cells and no obvious abnormalities. At week 6, compared with the blank group, some islet cells in group M showed necrosis, atrophy, pyknosis, and subsequent vacuolization. At weeks 10 and 14, pancreatic tissue in group M showed scattered inflammatory cells, interlobular edema, and interacinar edema. Compared with group M, the pathological changes in group J were milder. Cell necrosis, atrophy, pyknosis, vacuolization, inflammation, and edema were alleviated to varying degrees. These results indicated that long-term feeding with a high-sugar and high-fat diet caused serious pancreatic tissue damage in mice. After intragastric administration of JS, pancreatic cell pathology was significantly alleviated, and vacuolar degeneration of acinar cells was reduced.

**Figure 6:**
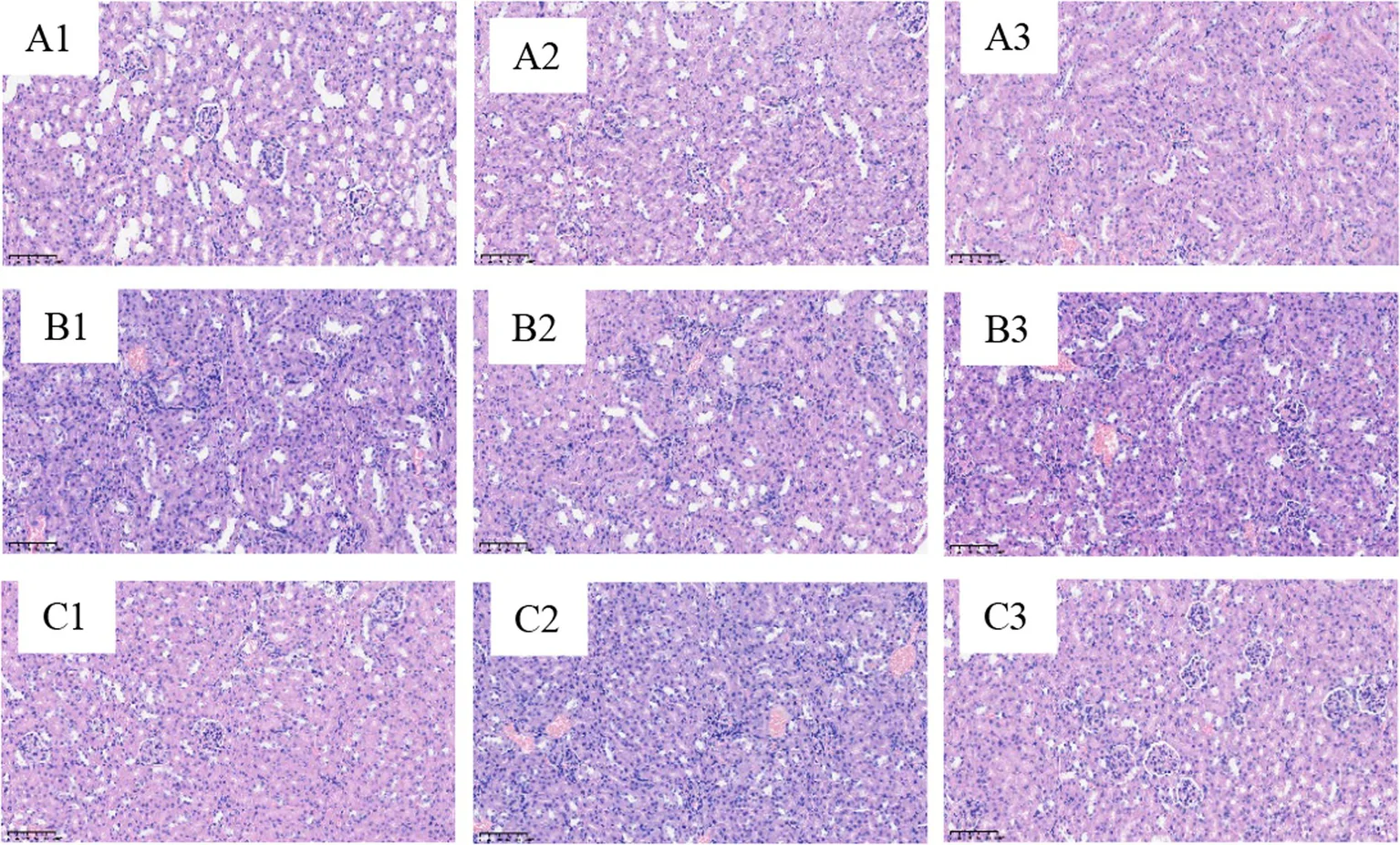
Representative H&E-stained renal sections at weeks 6, 10, and 14 (200×). N, normal control group receiving the basal diet and normal saline; M, HFD/STZ-induced hyperglycemic model group receiving normal saline; J, HFD/STZ-induced hyperglycemic group receiving LAB-fermented dandelion Jiangshui. A, B, and C denote N, M, and J, respectively; subscripts 1–3 denote weeks 6, 10, and 14.

Figure 6 showed that renal tissue in group N had a clear structure, normal morphology, a smooth basement membrane, regular glomeruli, no renal interstitial abnormalities, neatly arranged renal tubules, and no inflammatory cell infiltration. In contrast, the glomeruli in group M were enlarged, the basement membrane was thickened, obvious inflammatory cell infiltration was observed, and the renal tubules were slightly dilated. Compared with group N, pathological damage in group J was reduced, with only mild inflammatory cell infiltration and recovery of the renal tubules.

### 3.10 Changes in intestinal microorganisms

#### 3.10.1 Alpha diversity analysis of intestinal microflora in mice

Referring to the data in Table 7, compared with the control group, the Chao index and Shannon index of the intestinal microorganisms of the mice in group M were significantly lower than those in group N, while the Simpson index was significantly increased (*p < 0.05*). This result indicated that a long-term high-sugar and high-fat diet led to a decrease in the alpha diversity of the intestinal tract of the mice. Further comparison of the data between group M and group J showed that the Chao index and Shannon index of group J showed an increasing trend, while the Simpson index decreased. This indicated that after long-term intragastric administration of JS, the intestinal microecological state of the mice improved.

**Table 7:** Alpha diversity of intestinal flora in mice.

Groups	Chao1 index	Shannon index	Simpson index
N	567 ± 367^a^	4.15 ± 0.23^a^	0.026 ± 0.004^c^
M	283 ± 103^c^	2.42 ± 0.19^c^	0.208 ± 0.033^a^
J	419 ± 112^b^	3.52 ± 0.08^b^	0.103 ± 0.017^b^

N, normal control group receiving the basal diet and normal saline; M, HFD/STZ-induced hyperglycemic model group receiving normal saline; J, HFD/STZ-induced hyperglycemic group receiving LAB-fermented dandelion Jiangshui. Data are presented as the mean ± SD (*n* = 5 mice per group). Different lowercase letters in the same column indicate significant differences among groups (one-way ANOVA followed by the LSD test, *p* < 0.05).

#### 3.10.2 Species richness analysis

Based on the results of species annotation, the species with the top 9 abundances at the phylum level and the top 10 abundances at the genus level in the samples were screened out, and a stacked bar chart of species relative abundances was constructed.

**Figure 7:**
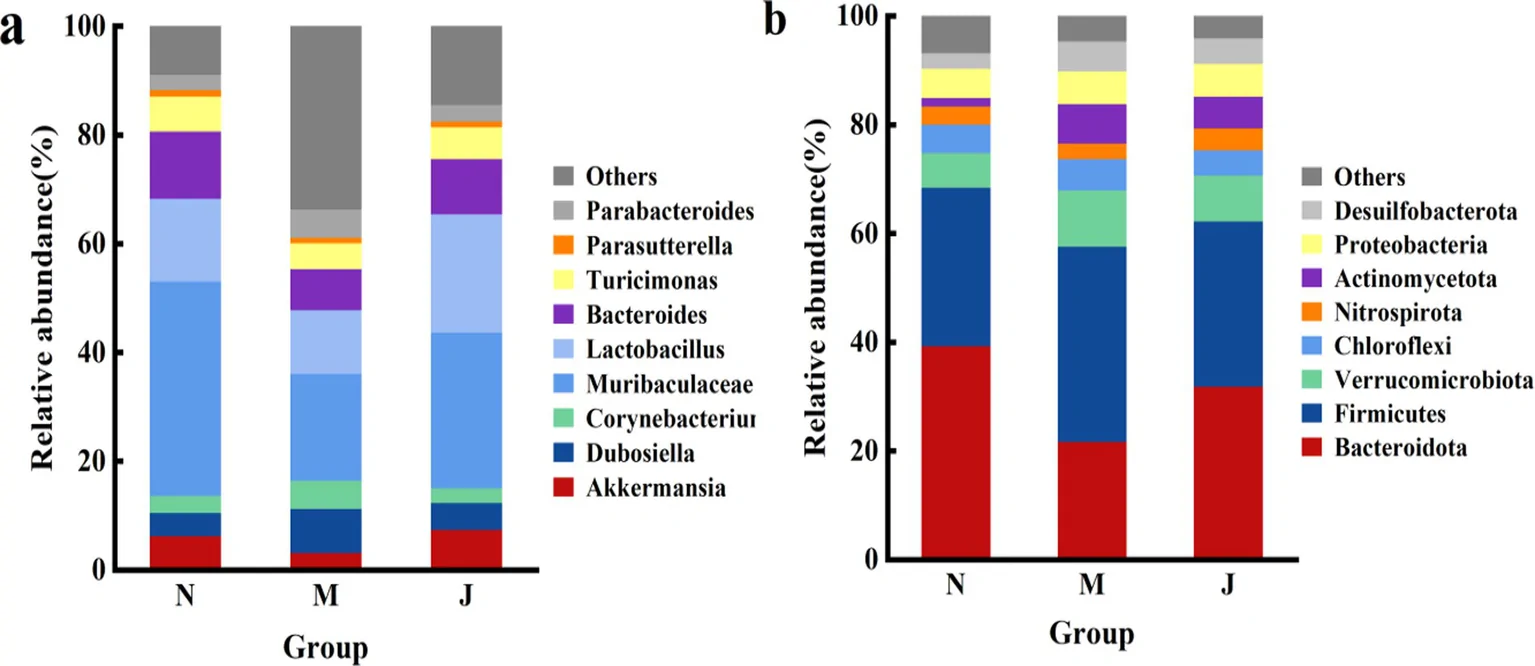
Relative abundance of gut microbiota. **(A)** Genus-level relative abundance; **(B)** phylum-level relative abundance. N, normal control group receiving the basal diet and normal saline; M, HFD/STZ-induced hyperglycemic model group receiving normal saline; J, HFD/STZ-induced hyperglycemic group receiving LAB-fermented dandelion Jiangshui. Bars show the mean relative abundance of five mice per group; low-abundance taxa are grouped as “Others”.

An analysis of the bar chart of bacterial abundances at the phylum level in Figure 7B showed that, compared with group N, the proportion of *Firmicutes* in the mice of group M increased, the proportion of *Bacteroidota decreased*, and the abundance of *Verrucomicrobiota increased*. When group J was compared with group M, the proportions of various phyla showed a certain degree of readjustment. Analysis of Figure 7A at the genus level showed that the relative abundance of *Akkermansia* in group J was significantly increased. As a potential probiotic in the intestine, *Akkermansia* can strengthen intestinal barrier function, reduce the permeability of the intestinal mucosa, and inhibit the inflammatory response. By reducing glucose absorption, regulating energy metabolism and other pathways, it can effectively improve insulin sensitivity and regulate blood glucose levels (Rodrigues et al., 2022).

## 4 Discussion

Fermented plant foods have attracted increasing research interest for their potential role in improving glucose and lipid metabolism in metabolic disorders (Li K. J. et al., 2022). Carrot juice fermented with *Lactiplantibacillus plantarum* NCU116 improved fasting glucose and insulin-related indices in HFD/STZ-induced diabetic rats, while *Lacticaseibacillus paracasei*-fermented *Rosa roxburghii* juice and probiotic-fermented tomato produced favorable changes in glucose and lipid metabolism in diabetic or HFD-fed mice (Li et al., 2014; Wei et al., 2023, 2024). Consistent with these studies, mice receiving LAB-fermented dandelion Jiangshui showed partial recovery of body-weight gain (Figure 2), lower fasting blood glucose (Table 4), improved glucose tolerance (Table 5), and lower HbA1c and insulin levels together with higher hepatic glycogen content than the model group (Table 6). The coordinated changes in short-term glycemic responses, longer-term glycemic exposure, circulating insulin, and hepatic glycogen suggest an overall improvement in glucose handling and storage rather than an isolated effect on one metabolic endpoint. These results therefore extend previous evidence from other fermented plant products and support the potential of LAB-fermented dandelion Jiangshui to modulate glucose homeostasis.

To further contextualize these metabolic responses, the phenolic changes in the fermented dandelion Jiangshui matrix were considered. LAB fermentation can alter the phenolic composition of plant matrices through microbial enzymatic activities (Gaur and Gänzle, 2023). LC–MS analysis showed that chlorogenic acid, caffeic acid, and isochlorogenic acids A and C became detectable after 36 h of fermentation, while luteoloside increased from 52.59 to 89.33 μg/L (Table 3). Chlorogenic acid showed the highest concentration among the five selected phenolic compounds in the fermented sample. Similar changes in phenolic composition have been reported for LAB-fermented dandelion products (Kim and Baik, 2015; Zhu et al., 2024). Collectively, these results provide matrix-level evidence that LAB fermentation altered the detectable phenolic composition of dandelion Jiangshui and offer compositional context for the responses observed in mice.

Beyond the changes in glucose metabolism, inflammation and oxidative stress are closely involved in hyperglycemia-associated pancreatic *β*-cell dysfunction and tissue injury (Chen et al., 2017; Dludla et al., 2023). *Limosilactobacillus fermentum* MF423-fermented rice bran was previously reported to reduce glucose-related abnormalities, enhance antioxidant activity, and alleviate hepatic lesions in diabetic mice (Ai et al., 2021). The J group exhibited a higher IL-10 level at week 14 (Figure 3), increased hepatic GSH-Px and CAT activities and lower MDA content (Figure 4), and milder pancreatic and renal lesions than the model group (Figures 5,6) (Sali et al., 2025). Although TNF-*α* and IL-6 varied transiently at week 10 and SOD activity did not change uniformly across all time points, the overall pattern indicates a time-dependent attenuation of inflammatory and oxidative imbalance. Persistent oxidative imbalance can promote lipid peroxidation, mitochondrial dysfunction, and inflammatory signaling, thereby contributing to diabetes-related tissue injury (Yaribeygi et al., 2020; Wang and Zhang, 2024). Accordingly, the lower hepatic MDA content together with higher GSH-Px and CAT activities may have accompanied the milder pancreatic and renal lesions observed in the J group (Warren et al., 2019). Overall, these findings indicate that the metabolic effects of fermented dandelion Jiangshui were accompanied by coordinated changes in inflammatory status, antioxidant defense, and tissue injury.

In addition to these systemic and tissue-level responses, LAB-fermented dandelion Jiangshui was also associated with changes in the gut microbial community. Gut microbiota remodeling is increasingly recognized as a feature accompanying metabolic improvements induced by probiotic and fermented-food interventions. Previous studies have associated *Akkermansia*, particularly *A. muciniphila*, with metabolic homeostasis and have reported parallel changes in gut microbial composition and glucose-related indices after fermented-plant interventions (Zhang et al., 2019; Rodrigues et al., 2022; Wei et al., 2023). The J group showed higher alpha-diversity indices (Table 7) and a higher relative abundance of *Akkermansia* than the model group (Figure 7), together with improvements in several glycemic, inflammatory, and oxidative stress indicators. The parallel occurrence of these changes suggests that LAB-fermented dandelion Jiangshui was accompanied by a shift toward a more diverse microbial community and enrichment of a genus frequently associated with metabolic health. Because the present observation is based on genus-level relative abundance, it is interpreted as an association rather than evidence that *Akkermansia* directly mediated the metabolic response. Overall, the microbiota findings complement the systemic and histological results and indicate that microbial community changes may form part of the broader biological response to fermented dandelion Jiangshui.

## 5 Conclusion

In conclusion, LAB-fermented dandelion Jiangshui prepared with A1: A3: L2 (1:1:1, v/v/v) was associated with moderated fasting blood glucose elevation, improved glucose tolerance and selected glucose-related indicators, attenuation of inflammatory and oxidative imbalance, less pronounced pancreatic and renal lesions, and changes in gut microbial composition in HFD/STZ-induced hyperglycemic mice. These findings support the potential development of LAB-fermented dandelion Jiangshui as a functional fermented food for glucose management. Although the absence of a standard drug control precluded direct comparison with established pharmacological treatment, the coordinated changes across glycemic, inflammatory, oxidative, histological, and gut microbiota-related outcomes provide preliminary evidence supporting further evaluation of LAB-fermented dandelion Jiangshui. The study was also limited to a single animal model and intervention design, and the underlying molecular mechanisms were not directly verified. Further studies incorporating an appropriate positive-control drug, additional models, broader dose ranges, and targeted mechanistic analyses are needed to confirm these findings and clarify their biological basis.

## Data Availability

The datasets supporting the findings of this study are available from the corresponding author upon reasonable request. Requests should be directed to guiyanwen@mail.lzjtu.cn.
